# Alcohol, Binge Drinking and Associated Mental Health Problems in Young Urban Chileans

**DOI:** 10.1371/journal.pone.0121116

**Published:** 2015-04-01

**Authors:** Amanda J. Mason-Jones, Báltica Cabieses

**Affiliations:** 1 Department of Health Sciences, University of York, York, England, United Kingdom; 2 Adolescent Health Research Unit, University of Cape Town, Cape Town, South Africa; 3 Universidad del Desarrollo, Santiago, Chile; Queensland University of Technology, AUSTRALIA

## Abstract

**Objective:**

To explore the link between alcohol use, binge drinking and mental health problems in a representative sample of adolescent and young adult Chileans.

**Methods:**

Age and sex-adjusted Odds Ratios (OR) for four mental wellbeing measures were estimated with separate conditional logistic regression models for adolescents aged 15-20 years, and young adults aged 21-25 years, using population-based estimates of alcohol use prevalence rates from the Chilean National Health Survey 2010.

**Results:**

Sixty five per cent of adolescents and 85% of young adults reported drinking alcohol in the last year and of those 83% per cent of adolescents and 86% of young adults reported binge drinking in the previous month. Adolescents who reported binging alcohol were also more likely, compared to young adults, to report being always or almost always depressed (OR 12.97 [95% CI, 1.86-19.54]) or to feel very anxious in the last month (OR 9.37 [1.77-19.54]). Adolescent females were more likely to report poor life satisfaction in the previous year than adolescent males (OR 8.50 [1.61-15.78]), feel always or almost always depressed (OR 3.41 [1.25-9.58]). Being female was also associated with a self-reported diagnosis of depression for both age groups (adolescents, OR 4.74 [1.49-15.08] and young adults, OR 4.08 [1.65-10.05]).

**Conclusion:**

Young people in Chile self-report a high prevalence of alcohol use, binge drinking and associated mental health problems. The harms associated with alcohol consumption need to be highlighted through evidence-based prevention programs. Health and education systems need to be strengthened to screen and support young people. Focussing on policy initiatives to limit beverage companies targeting alcohol to young people will also be needed.

## Introduction

The WHO ‘Global strategy to reduce the harmful use of alcohol’[1] advocates for a clearer understanding of harmful alcohol use in every country and particularly in emerging economies. These countries, whilst promising huge potential for economic growth can simultaneously pose significant political, social and health risks to their populations [2]. For example, there is evidence that young people, especially those in urban areas, are at increased risk in these economies, from harmful alcohol use [3]. Despite this, the problem of youth alcohol drinking remains an under-researched phenomenon [4]. This is concerning because of the evidence emerging about the links between mental health problems related to alcohol use in young people [5]. For example, among those in the 10–24 age group worldwide, the main cause for years lived with disability were mental health disorders [6] and the main risk factor was alcohol. It has been recognised that adolescents and young adults are an especially vulnerable population with regard to problematic alcohol consumption[7]. Possible explanatory factors suggested for the link between alcohol consumption and mental health problems are discrimination [8], concomitant drug consumption [9], problematic family functioning [10], sex, age, and general wellbeing [11]. In Chile, loneliness, impulsivity and alcohol use have also been highlighted as risk factors for suicide among adolescents [12]. Despite the evidence, alcohol beverage companies are allowed to continue to target youth [13] and often dispute the prevalence of alcohol-related problems [14,15].

Alcohol use is one of the leading causes of both mortality and morbidity in Latin America [16] and is considered to be a regional public health threat requiring urgent action [17]. Chile, with a population of just over 16 million [18], has a largely urban population (over 88% live in cities) who have experienced extensive economic changes in recent years [19]. The country is regarded as a ‘global player’; Indeed, Chile became a high-income OECD member country in 2011 [20]. This rapid socioeconomic transformation has, along with some gains, resulted in significant pressures for young people. Chile has a rising middle class with disposable income, a large young population and an informal alcohol sector. Moreover, alcohol has been identified as the largest risk factor for death and disability in Chile [21] but there remains very limited information about alcohol use in young people in Chile. One of the few studies, a school-based study of adolescents, found that students in Grade 9 (14 years old), 38.8% reported drinking alcohol in the previous 2 weeks [22]

The aim of this study was to find out more about the association between harmful alcohol use and mental health problems measured by self-reported anxiety and depression and medical diagnosis for depression from a representative sample of young people in Chile using the Chilean Health Survey 2010.

## Materials and Methods

Since 2000, the Chilean Ministry of Health has made efforts to obtain information about population´s health through national health surveys [19]. We utilised the Chilean National Health Survey 2010 (ENS 2010) as a source of data for our study with approval granted by the Ministry of Health in Chile to access the anonymised dataset from a secured governmental website (http://epi.minsal.cl/estudios-y-encuestas-poblacionales/encuestas-poblacionales/). Any researcher can access this dataset after completing a brief on line questionnaire. However, neither the Ministry nor the funding institutions had any role in the design, execution, analysis or decision to publish these data.

### Data collection

The cross-sectional survey used a random multistage sampling of households (stratified by urban/rural location) with national and regional representation. The target population were those aged 15 years and older. The ENS survey employs multistage probabilistic sampling with two phases (county and household), stratified by urban/rural. The sampling frame included all regions in Chile from over 600 unique geo referenced counties created by the national institute of statistics in 2006. Around 20 hard to reach counties were excluded. Within each county, households were randomly selected. This complex sampling strategy allows the creation and use of sample weights in analysis, in order to attain to population-based estimates.

Data collection was via face-to-face interview by trained interviewers, using a validated questionnaire. The preferred respondent for the household socio-demographics was the reported head of household, followed by their spouse or an adult household member. For questions on health status and health outcomes, all household members of 15 years of age or older were asked to complete the questionnaire individually. The response rate was 85% and after the recruitment phase, 5 293 people were interviewed. Data for two specific age groups relevant to this study were extracted: for adolescents (15 to 20 years of age) and young adults (21 to 25 years of age).

### Outcome measures

The following dependent variables were used in the models:

1. Self-reported general life satisfaction: ‘how would you rate your life in general in the last year’? It allowed for the following response categories: very good, good, regular, bad, very bad. We recoded the 5 categories into two: poor (“1”: bad and very bad) and fair/good (“0”: regular, good and very good).
2. Depressed in the last month: ‘how often did you feel depressed last month’? Response categories were ‘always/ almost always’, ‘occasionally’, and ‘rarely/never’. We analysed this variable as collected for descriptive purposes first and then recoded it into high depressive symptoms (“1”: always/ almost always) and low/no depressive symptoms (“0”: occasionally, and rarely/never) for the multiple regression.
3. Anxious in the last month: ‘how anxious did you feel last month’? Response categories were ‘very’, ‘moderately’ and ‘not at all’. We analysed this variable as collected for descriptive purposes first and then recoded it into highly anxious (“1”: very) and not/moderately anxious (“0”: occasionally, and rarely/never) for multiple regression.
4. Depression diagnosis: ‘have you ever been diagnosed with depression’? Possible answers were ‘yes’ (labeled as “1”) or ‘no/don´t know’ (labeled as “0”).

The following independent variables related to alcohol consumption:

1. Alcohol prevalence in the last year: ‘Have you consumed any alcohol in the last year’? Response ‘yes’ was labeled as “1” and ‘no’ was labeled as “0”.
2. Binge drinking prevalence last month: ‘Did you binge-drink alcohol at least once last month’? The definition of this was if they had drunk four or more units of alcohol in a single episode in the last 4 weeks. Those reporting ‘yes’ were labeled as “1” and those reporting ‘no’ were labeled as “0”.

The “units of alcohol” indicator represented the number of self-reported 200 ml glasses of alcohol consumed. This was as a standard measure adapted from one of the 10 items of the "Alcohol Use Disorders Identification Test" (AUDIT) questionnaire developed by the World Health Organization (WHO) [23] and adapted for use in Chile [24].

We included age, sex, urban/rural residency, type of healthcare provision, and tobacco consumption as additional control variables. Age was included as a continuous variable for each age group under study whilst sex (male/female), urban/rural residence and type of health care provision (public, private, and other or don’t know) were also included as categorical variables. Tobacco consumption was asked as the following question: ´Have you ever smoked in your life?´ Possible answers were ´never´ (labeled as “0”), ´former smoker´ (labeled as “1”), and ´current smoker´ (labeled as “2”).

### Statistical analysis

Descriptive statistics for dependent and independent variables were reported as means (continuous variable) and proportions (categorical variables) with their 95% confidence intervals. All analyses for adolescents and young adults were conducted separately in order to explore if there were distinctive patterns of behaviour and mental wellbeing in these different age groups. In order to estimate the direction of magnitude of existing associations between behaviours and mental wellbeing, we estimated age and sex-adjusted Odds Ratios (OR) for each of the four mental wellbeing measures. Conditional logistic regression models (for each age group separately) were estimated by demographics, and alcohol consumption. For covariates with more than two categories (e.g. healthcare provision), we tested overall’s statistical signification through the adjusted Wald test and reported it as significant in the final tables (a p-value <0.05 represented a significant overall association between the variable and the outcome of interest). With regard to post-estimation tests, the Archer and Lemeshow goodness of fit test for a logistic regression model fitted using survey sample data was estimated (F-adjusted mean residual GOF test) [25]. Data analyses were conducted with the STATA 12 statistical software package [26] and estimations were weighted to take into account the complex multistage sampling strategy of the survey [27].

The ENS survey included an exhaustive list of health problems and health related behaviors. A full description of the measures used in the survey are freely available in the Ministry of Health Web page (http://epi.minsal.cl/estudios-y-encuestas-poblacionales/encuestas-poblacionales/encuesta-nacional-de-salud/cuestionarios-ens/). Before final analysis, we explored the association between such health conditions and mental health problems (main outcome of this study). This analysis is fully available in S1 Table. We found no strong or consistent association between mental wellbeing and such infectious or chronic health problems in our study samples; hence, these have not been included here.

## Results

The population sample of 435 adolescents and 412 young adults represented 1,860,812 and 1386,547 respectively. There was a balance between males and females, most (88%) lived in urban settings and the vast majority used the public health care system (85% of adolescents and 72% young adults) rather than private health care. Nine per cent of adolescents and 10% of young adults reported always or almost always feeling depressed in the previous month and 21% of adolescents and 28% young adults reported feeling occasionally depressed in the last month. Sixty five per cent of adolescents and 85% of young adults reported drinking alcohol in the last year and 83% of adolescents and 86% of young adults reported binge drinking alcohol at least once in the previous month. (Table 1).

**Table 1:** Demographic characteristics, mental wellbeing and reported alcohol consumption in adolescents and young adults, Chilean National Health Survey 2010.

	Adolescents (15–20 years old) Proportion % [95%CI] Absolute n = 435 (Weighted n = 1 860 812)	Young adults (21–25 years old) Proportion % [95%CI] Absolute n = 412 (Weighted n = 1 386 547)
*DEMOGRAPHIC CHARACTERISTICS*		
Sex (female)	49.71 [42.93–56.50]	49.17 [41.05–57.34]
Rural setting	11.40 [7.91–16.17]	12.66 [8.59–18.29]
Healthcare provision:		
Public	84.45 [79.22–88.55]	72.20 [63.04–79.83]
Private	11.06 [7.70–15.64]	25.76 [18.29–34.97]
Other or don´t know	4.49 [2.41–8.21]	2.04 [0.63–6.34]
*MENTAL WELLBEING*		
Self-reported general life satisfaction (poor)	1.86 [0.92–3.72]	2.91 [1.36–6.08]
How often did you feel depressed last month?		
Always/ almost always	8.73 [5.78–12.98]	9.68 [6.28–14.65]
Occasionally	20.89 [16.34–26.31]	27.37 [21.21–34.53]
Rarely/ never	70.37 [64.28–75.82]	62.95 [55.22–70.07]
How anxious did you feel last month?		
Very	12.68 [8.93–17.77]	21.42 [15.84–28.32]
Moderately	16.98 [12.61–22.44]	21.36 [15.70–28.38]
Not at all	70.33 [63.94–76.02]	57.21 [49.12–64.93]
Ever diagnosed with depression? (yes)	10.49 [7.38–14.70]	22.05 [16.24–29.22]
*ALCOHOL CONSUMPTION*		
Alcohol consumption last year (yes)	65.29 [58.58–71.45]	84.72 [78.74–89.26]
Binging alcohol at least once last month (yes)	83.30 [72.85–90.27]	85.69 [78.74–90.63]
*TOBACCO CONSUMPTION*		
Never	48.86 [42.08–55.69]	30.75 [24.09–38.40]
Former smoker	40.63 [34.05–47.55]	60.18 [52.22–67.64]
Current smoker	10.51 [7.36–14.79]	9.07 [5.93–13.62]

Adolescents who reported binge drinking alcohol were also more likely to have reported feeling always or almost always depressed in the last month (OR 12.97, 95% CI, 1.86–21.09) or very anxious in the previous month (OR 9.37, 95% CI, 1.77–19.54) while young adults were more likely to report always or almost always feeling depressed in the last month (OR 1.52, CI, 1.07–2.16) compared to adolescents. Compared to adolescents that never smoked, former smokers reported a higher chance of ever been diagnosed with depression (OR 2.90, 95%CI 1.14–33). (Table 2).

**Table 2:** Odds Ratio (ORs)^ of presenting low wellbeing by alcohol risk behaviours, measured by general life satisfaction, anxiety and depression symptoms or diagnosis, in adolescents and young adults, Chilean National Health Survey 2010.

	Adolescents (15–20 years old)				Young adults (21–25 years old)			
	Poor self- reported general life satisfaction OR [95%CI]	Always/almost always felt depressed last month OR [95%CI]	Very anxious last month OR [95%CI]	Ever diagnosed with depression OR [95%CI]	Poor self- reported general life satisfaction OR [95%CI]	Always/almost always felt depressed last month OR [95%CI]	Very anxious last month OR [95%CI]	Ever diagnosed with depression OR [95%CI]
Age	***0.69 [0.47–1.00]*** *	1.04 [0.83–1.29]	1.04 [0.8–1.31]	0.88 [0.69–1.11]	1.10 [0.78–1.54]	***1.52 [1.07–2.16]*** *	1.04 [0.78–1.38]	0.81 [0.60–1.08]
Sex (female)	***8.50 [1.61–15.78]*** **	***3.41 [1.25–9.58]*** **	1.51 [0.63–3.62]	***4.74[1.49–15.08]*** **	0.62 [0.13–2.86]	1.50 [0.58–3.87]	1.48 [0.66–3.32]	***4.08 [1.6–10.05]*** **
*ALCOHOL CONSUMPTION and BINGE DRINKING ALCOHOL*								
Alcohol consumption last year (yes)	1.40 [0.36–5.99]	1.30 [0-51-3.33]	0.88 [0-38-2.05]	0.59 [0.26–1.36]	0.75 [0.10–5.51]	0.20 [0.69–7.00]	1.38 [0.54–3.51]	1.03 [0.43–2.48]
Binging alcohol at least once last year (yes)	0.52 [0.05–1.77]	***12.97 [1.86–21.09]*** **	***9.37[1.7–19.54]*** **	3.12[0.56–7.17]	3.34 [0.93–12.32]	2.82 [0.51–15.51]	0.91 [0.30–2.70]	0.96 [0.27–3.36]
*TOBACCO CONSUMPTION*								
Never (ref.)	1.00	1.00	1.00	1.00	1.00	1.00	1.00	1.00
Former smoker	0.87 [0.22–3.43]	1.05 [0.38–3.21]	0.55 [0.29–1.05]	***2.90 [1.14-.33]*** *	0.94 [0.19–4.63]	0.94 [0.28–3.37]	0.86 [0.41–1.78]	2.10 [0.84–5.26]
Current smoker	0.86 [0.10–7.83]	1.34 [0.33–5.43]	0.69 [0.29–2.99]	2.13 [0.67–6.68]	No cases	0.53 [0.08–3.47]	0.69 [0.24–2.00]	0.70 [0.21–2.52]

*p-value <0.05

** p-value <0.01

^^^ Each OR adjusted by sex and age

Most variables of interest appeared to be balanced between males and females in both study groups, with a few distinctive features. For example adolescent girls were more likely to report poor general life satisfaction than boys (3.2% versus 0.46%), but this reverted in the young adults´ group in which males reported poorer life satisfaction than females (3.3% versus 2.4%). In both study groups, females reported a higher proportion of feeling always or almost always depressed in the last month, as well as feeling very anxious during the same time period. Females in both study groups were 2 to 3 times more likely to report ever been diagnosed with depression than males, and more likely to report being a current smoker. However, males were more likely to report drinking alcohol and binging alcohol than females (Table 3).

**Table 3:** Demographic characteristics, mental wellbeing and reported alcohol and tobacco consumption of the study sample by sex, Chilean National Health Survey 2010.

	Adolescents (15–20 years old) Proportion % [95%CI]		Young adults (21–25 years old) Proportion % [95%CI]	
*DEMOGRAPHIC CHARACTERISTICS*	*MALES*	*FEMALES*	*MALES*	*FEMALES*
Rural setting	12.15 [6.89–20.53]	10.65 [6.75–16.41]	9.84 [4.59–19.83]	15.59 [10.25–22.98]
Healthcare provision:				
Public	83.41 [74.42–87.87]	85.51 [79.19–90.14]	62.90 [48.64–75.22]	81.82 [71.70–88.88]
Private	9.08 [3.42–16.78]	13.06 [8.60–19.39]	34.87 [22.71–49.39]	16.34 [9.66–26.28]
Other or don´t know	7.51 [3.64–14.87]	1.44 [0.62–3.31]	2.22 [0.22–10.86]	1.84 [0.36–8.98]
*MENTAL WELLBEING*				
Self-reported general life satisfaction (poor)	0.46 [0.11-1-96]	3.28 [1.51–6.32]	3.38 [1.10–9.90]	2.42 [0.96–5.42]
How often did you feel depressed last month?				
Always/ almost always	4.13 [1.78–9.27]	13.40 [8.42–20.65]	7.72 [3.74–15.26]	11.72 [6.83–19.37]
Occasionally	17.78 [11.45–26.56]	24.04 [18.10–31.19]	26.20 [17.20–37.76]	28.58 [20.91–37.72]
Rarely/ never	78.10 [68.97–85.12]	62.56 [54.27–70.27]	66.78 [54.02–76.37]	59.71 [49.91–68.88]
How anxious did you feel last month?				
Not at all	73.52 [63.26–81.43]	67.11 [58.90–74.40]	64.08 [51.72–74.82]	50.11 [40.02–62.31]
Moderately	16.58 [10.15–25.91]	17.39 [12.14–24.30]	18.46 [11.30–28.68]	24.37 [16.40–34.21]
Very	9.90 [10.15–25.91]	15.49 [10.35–22.55]	17.46 [10.05–28.60]	25.52 [18.06–34.56]
Ever diagnosed with depression? (yes)	4.11 [1.43–11.25]	16.94 [11.92–23.51]	10.96 [5.56–20.46]	33.43 [24.34–43.94]
*ALCOHOL CONSUMPTION*				
Alcohol consumption last year (yes)	72.34 [62.11–81.58]	57.56 [49.02–65.66]	90.10 [80.03–95.39]	79.16 [79.98–85.64]
Binging alcohol at least once last month (yes)	89.07 [74.94–95.69]	70.83 [52.34–83.40]	92.74 [85.57–96.49]	71.10 [56.37–82.41]
*TOBACCO CONSUMPTION*				
Never	47.33 [36.87–58.03]	50.41 [41.93–58.88]	27.33 [18.18–38.91]	34.31 [25.28–44.63]
Former smoker	46.94 [36.48–57.67]	54.44 [26.65–42.72]	64.30 [54.10–74.89]	55.90 [45.70–65.62]
Current smoker	5.73 [2.85–11.34]	15.35 [10.25–22.34]	8.37 [4.00–16.67]	9.80 [6.09–15.40]

Overall, females were significantly more likely than males to report poor life satisfaction (OR 8.50, 95% CI, 1.61–15.78) and to report that they had been diagnosed with depression (OR 4.74, 95% CI, 1.49–15.08). Goodness if fit statistics of regression models were all adequate according to the Archer and Lemeshow survey GOF estimate, with all p values above significance.

## Discussion

We explored the prevalence of both alcohol use and mental health problems in a representative sample of adolescents and a young adult population in Chile from the Chilean National Health Survey 2010. Our findings suggest that adolescents and young adults report high levels of binge drinking, significant mental health problems and a relationship between these two factors. The prevalence of harmful alcohol use that we found is much higher than that reported in a previous school-based study of this population in Chile [22]. How the complex relationship between alcohol use, particularly binge drinking, and mental health operates is not clear but this link has also been confirmed in other studies [28]. This association has been variously seen as related to shared risk factors between harmful alcohol use and mental health issues [29], alcohol-induced depression which is different from other major independent depressive disorders [30] or stress that leads to increased alcohol consumption and binge drinking [31]. Clearly further work is needed to clarify these multiple interconnected pathways. Either way we found that there is a significant proportion of mental health problems among young people not being picked up by health services particularly those of young men; also confirmed by recent published work which highlights this as a global issue [32,33]. This is, to our knowledge, the first study to document these issues in Chile and highlights this as a potential issue for other rapidly emerging economies.

In our analysis, we found little association between tobacco consumption and mental health problems. Compared to late adolescents that never smoked, former smokers reported a higher chance of ever been diagnosed with depression (OR 2.90, 95%CI 1.14-.33). Additionally, we explored if our main mental health problems and explanatory measures were associated with other infectious or chronic health problems available in the ENS 2010 dataset. However, we could not find any consistent correlation across such measures, perhaps due to the young age of the groups under study and the high prevalence of chronic conditions in the general Chilean population, largely from 40 years of age onwards (S1 Table).

This study is based on a nationally representative sample in Chile but has some limitations. We acknowledge that we rely on self-reports of behaviour and mental wellbeing such that the reports of drinking could have been overestimated and mental health difficulties underestimated or vice versa. Also, this study is a cross-sectional design and simply highlights the strong association between risky alcohol use and mental wellbeing in young people in Chile and because it was aimed at the general adult population, we do not have any information about adolescents younger than 15 years of age. Our estimates were imprecise with wide confidence intervals suggesting that we need a larger sample and because the ENS 2010 does not adequately assess socioeconomic inequality we were unable to include it in the model. Despite the fact that all GOF test estimates were adequate in these analyses, we are cautious of any causal interpretation and we define this study as exploratory and ‘hypotheses generating’. Many possibly relevant variables were not available; however, we exhausted possibilities by adding significant control variables like socio-demographics, healthcare provision and tobacco consumption in our analyses, as well as exploring correlations between mental health indicators and several chronic and infectious diseases.

One additional limitation of our analysis was converting the three-category variables of anxiety and depressive symptoms into binary ones. We attempted to conduct multinomial regressions for these, but due to small sample sizes, regression estimates were not parsimonious and they lacked of statistical fit. Therefore, we chose the conservative alternative of pulling together those of “moderate” symptoms with the no symptoms. This decision allowed us to explore in a more robust fashion the “very” anxious category, the generally accepted more vulnerable group, at the risk of not being able to comment on the “moderate” groups.

Chile’s relationship with alcohol changed following Spanish colonization in the XV century and became more a part of social life [34]; As in many countries, prior to colonisation, alcohol and drugs were generally used for special religious purposes [35]. Today, drinking patterns continue to change. Recent work by Sanhueza and colleagues [36] suggests that alcohol initiation often takes place around ‘fiesta’ and the socio-cultural context supports the high prevalence of alcohol use in that it ameliorates daily-life struggles and that binge drinking is a particular problem. Indeed, adult per capital alcohol consumption within 68 countries has been strongly associated with adolescent alcohol use (r = 0.81, P<0.001) [37]. This may be as a result of shared risk factors and predictors may differ from place to place. The conclusions from our data are also supported by other studies. A study analysing the Youth Health Risk Behaviour Survey in Brazil [38] found that adolescents who were considered to be ‘episodic heavy drinkers’ i.e. those that had five or more drinks in a 2-hour interval were also more likely to be older and male and that those in public schools were more likely to have attempted suicide (OR, 4.2, CI, 2,8.7) than moderate drinkers. Another study using data from the Global School-based Health Survey (GSHS) found that scoring high on the ‘global’ psychological distress (loneliness, worrying, feeling sad/hopeless, having a suicide plan) was statistically significantly related to substance use including alcohol, for both male and female substance users compared to non-substance users in the Philippines, China, Chile and Namibia. The authors suggest that this suggests a ‘relationship (*between psychological distress and substance abuse*) that is more universal than context specific’ p.919 [39]

It has also been demonstrated that there is an association between alcohol marketing and the increasing uptake and frequency of drinking in young people [40]. Certainly alcohol companies target emerging economies which provide new opportunities [41] and particularly in sub-Saharan Africa with its large young population has found that patterns of alcohol consumption have increased [42]. Beer-drinking particularly is more closely tied to economic growth than spirits and is frequently the choice of alcohol for young people [43]. However this can be problematic for increasingly harmful use of alcohol. Bennet and colleagues [44] in a study that included India, Nigeria and Mexico found that whilst there were cultural differences, emerging markets were prime targets for drinks companies, and young people in all of these countries were binge drinking alcohol.

The “Chile Crece Contigo” [45] initiative recognises the importance of living conditions to children’s health and development. Youth is a key period particularly for studying the development of mental health and wellbeing over the life course, with studies in Europe and North America showing a pattern of elevated risk of mental health problems such as depression that begin in early adolescence and that continue to across the life course [46]. Furthermore there are studies evidencing the high prevalence of alcohol consumption among adolescents in Chile and some individual socio-demographic and contextual social factors related to it [36]. However, little has been written about adolescent and young adult health and wellbeing in Chile [47,48]. Effective prevention and treatment strategies are essential for ensuring that any risks to health from alcohol use are minimised [49].

Increasing price and controlling sales, and restricting advertising and promotions that target youth may be the most effective intervention for regulating the context within which alcohol is consumed and reducing consumption and alcohol-related harm [50] as the evidence for the effectiveness of school-based and other interventions is limited [51,52]. Screening and treatment services also need to be in place for mental health including for adolescents at school [33] as evidence shows that it is very difficult to recover without this support[53,54]. Despite the burden of disease related to alcohol, particularly in low and middle income countries, and in poor populations in all countries, prevention measures need to be implemented alongside care for those with more developed alcohol use disorders [55]. Support and treatment for those with comorbidity of alcohol and mental health issues will in turn have benefits for these countries in the longer term. However, moving away from focusing on alcohol dependence and looking further at harmful/hazardous drinking behaviours may help to focus policy makers and planners on a framework for alcohol control.

## Conclusion

This study has shown how Chile, similarly to other emerging economies, may have unwittingly created new and potential risks of mental ill-health [56,57] and alcohol use [5] for their young populations during this period of rapid growth. Further research and political attention is needed to explore the relationship between mental health, alcohol use and binge drinking and limiting and restricting alcohol marketing to young people living in Chile and other emerging economies.

## Supporting Information

S1 Table Exploring associations (Odds Ratios, OR, and 95% Confidence Intervals, 95%CI) between mental health problems and infectious or chronic conditions from the ENS 2010 dataset for adolescents and young adults. (DOCX)
