# Supplementary material for: Alcohol, Binge Drinking and Associated Mental Health Problems in Young Urban Chileans
Source: PLoS One. 2015 Apr 1;10(4):e0121116. doi: 10.1371/journal.pone.0121116 (PMC4382020; doi:10.1371/journal.pone.0121116)
Supplement: S1 Table — (DOCX) [file pone.0121116.s001.docx]

**Table S1** Exploring associations (Odds Ratios, OR, and 95% Confidence Intervals, 95%CI)^ between mental health problems and infectious or chronic conditions from the ENS 2010 dataset for adolescents and young adults.

|  | **Adolescents (15-20 years old)** | | | | **Young adults (21-25 years old)** | | | |
| --- | --- | --- | --- | --- | --- | --- | --- | --- |
|  | **Poor self- reported general life satisfaction** OR [95%CI] | **Always/almost always felt depressed last month** OR [95%CI] | **Very anxious last month** OR [95%CI] | **Ever diagnosed with depression** OR [95%CI] | **Poor self- reported general life satisfaction** OR [95%CI] | **Always/almost always felt depressed last month** OR [95%CI] | **Very anxious last month** OR [95%CI] | **Ever diagnosed with depression** OR [95%CI] |
| Hypertension | No cases | No cases | ***9.75 [3.03-29.92]***** | No cases | No cases | No cases | No cases | No cases |
| Diabetes Mellitus | No cases | 2.04 [0.36-11.48] | 1.38 [0.18-13.66] | 0.27 [0.02-3.20] | No cases | 0.6 [0.1-1.12] | 0.06 [0.1-1.32] | 0.6 [0.1-1.12] |
| Obesity | 1.14 [0.18-8.18] | 2.87 [0.99-8.27] | 1.11 [0.41-2.87] | 0.55 [0.13-2.19] | 1.40 [0.25-7.82] | 1.28 [0.36-4.53] | ***0.37 [0.17-0.90]**** | 2.59 [0.83-8.09] |
| Metabolic Syndrome | No cases | 3.35 [0.47-13.59] | 0.51 [0.11-2.29] | No cases | No cases | 1.50 [0.31-7.07] | 0.81 [0.29-2.23] | 1.57 [0.44-5.54] |
| Hypercholesterolemia | 4.42 [0.49-19.99] | 1.87 [0.28-12.26] | 0.89 [0.25-3.17] | 1.36 [0.28-6.40] | 0.12 [0.01-1-16] | 2.12 [0.58-7.73] | 0.64 [0.23-1.75] | 0.85 [0.26-6-62] |
| Thyroid disease | 2.17 [0.18-17.26] | 0.21 [0.03-1.38] | 0.69 [0.29-2.36] | 2.37 [0.53-10.65] | 2.33 [0.37-14.38] | 1.25 [0.26-6.00] | 1.92 [0.63-5.80] | 1.78 [0.45-7.10] |
| Diarrhoea last year | 1.20 [0.22-6.44] | ***2.74 [1.04-7.24]**** | 1.51 [0.69-3.29] | 0.89 [0.36-2.17] | 3.55 [0.78-16.02] | 1.98 [0.68-9.54] | 1.30 [0.62-2.72] | 2.15 [0.19-5.09] |
| HIV infection | 0.23 [0.02-3.21] | 0.15 [0.02-1.10] | 1.30 [0.53-3.17] | 1.98 [0.74-12-34] | 3.03 [0.44-16.53] | 2.12 [0.78-8.43] | 0.90 [0.58.8.32] | ***2.63 [1.15-5.99]**** |

*p-value <0.05

** p-value <0.01

^ Each OR adjusted by sex and age
